# Admission CD4^+^ T cell BTLA expression and its association with infection risk and respiratory outcomes in sepsis

**DOI:** 10.3389/fcimb.2026.1852916

**Published:** 2026-08-03

**Authors:** Shuwei Ning, Ying Liu, Changmeng Cao, Ailin Song, Rongxin Sun, Ziqi Rong, Chungang Zhao

**Affiliations:** Department of Critical Care Medicine, Dalian Friendship Hospital Affiliated To Dalian Medical University, Geriatric Medical Center, Dalian, Liaoning, China

**Keywords:** BTLA, ICU-acquired infection, immune suppression, sepsis, ventilator weaning

## Abstract

**Objective:**

T cell co-inhibitory molecule upregulation is linked to sepsis-induced immune suppression, but the relationship between B and T lymphocyte attenuator (BTLA) expression and ICU-acquired infection or organ function recovery in sepsis remains unclear.

**Methods:**

This single-center prospective observational cohort study enrolled 143 septic ICU patients. BTLA on CD4+/CD8+ T cells was measured at ICU admission and on days 3/7. Patients were stratified into high- (n=72) and low- (n=71) BTLA-CD4 groups by median baseline CD4+ BTLA level. Primary outcome was ICU-acquired infection developing more than 48 hours after ICU admission. Secondary outcomes included PaO_2_/FiO_2_ ratio, ventilator weaning time, 28-day ventilator-free days and 28-day mortality.

**Results:**

The high-expression group had higher ICU-acquired infection (38.9% vs.21.1%, P = 0.033) and 28-day mortality (33.3% vs.9.9%, P = 0.001), but BTLA-CD4 was not an independent predictor for either after multivariable adjustment (infection: OR = 0.937, 95%CI:0.859–1.021, P = 0.139; mortality: HR = 1.034, 95%CI:0.965–1.108, P = 0.342). In a competing-risks analysis accounting for death before weaning, higher BTLA-CD4 was independently associated with delayed ventilator weaning (cause-specific HR = 0.882, 95%CI:0.832–0.936, P<0.001; Fine–Gray subdistribution HR = 0.940, 95%CI:0.902–0.981, P = 0.004). The low-expression group had faster oxygenation recovery and more ventilator-free days (P<0.001).

**Conclusion:**

Elevated CD4+ T cell BTLA expression at ICU admission is independently associated with delayed ventilator weaning in sepsis. Its links to infection and mortality appear to be driven by disease severity. Admission CD4+ T cell BTLA expression may identify a sepsis immune phenotype associated with impaired respiratory recovery, rather than acting as a direct regulator of lung repair.

## 1 Introduction

Sepsis represents a critical form of organ dysfunction that claims approximately 11 million lives annually, with an estimated 48.9 million new cases reported worldwide each year (Rudd et al., 2020). Despite continued advances in therapeutic strategies, mortality among septic patients remains substantially elevated. The development of ICU-acquired infections further compounds the disease burden and is independently associated with increased mortality risk (Vincent et al., 2009). Accumulating evidence indicates that sepsis patients may transition into an immunosuppression-dominant phase as early as the initial stages of illness, wherein T cell apoptosis and functional exhaustion emerge as critical determinants of secondary infections and late mortality (Hotchkiss et al., 2001; Hotchkiss et al., 2013). Co-inhibitory molecules expressed on the T cell surface have been closely implicated in immune dysfunction and adverse clinical outcomes (Chang et al., 2014; Patera et al., 2016).

B and T lymphocyte attenuator (BTLA) functions as an inhibitory receptor that attenuates T cell activation (Watanabe et al., 2003). Substantially upregulated BTLA expression on CD4^+^ T cells has been documented in septic patients in previous investigations, with such elevation linked to the later onset of nosocomial infections (Shubin et al., 2013). Substantial upregulation of BTLA and other co-inhibitory molecules has also been observed in splenic tissue obtained from patients who died of sepsis (Boomer et al., 2011). Furthermore, experimental evidence from animal models indicates that BTLA gene knockout improves survival in murine sepsis (Shubin et al., 2012).

However, existing clinical investigations have predominantly employed small-sample, cross-sectional designs, and systematic longitudinal characterization of BTLA expression dynamics remains lacking. Moreover, prior research has largely focused on the relationship between BTLA and infection or mortality, with limited attention given to its association with organ function recovery—particularly pulmonary recovery and liberation from mechanical ventilation. It also remains incompletely resolved whether the adverse outcomes accompanying elevated BTLA expression reflect an independent contributory role of BTLA itself or represent a concomitant marker of disease severity.

Therefore, 143 septic ICU patients were prospectively enrolled, and BTLA expression on CD4^+^ and CD8^+^ T cells was serially assessed at three predefined time points — within 24 hours of ICU admission, on day 3, and on day 7. This study primarily aimed to examine the relationship between baseline BTLA expression and ICU-acquired infection, and to assess whether this relationship persisted as an independent association following multivariable adjustment. Secondary objectives included characterizing the longitudinal trajectory of BTLA expression, and assessing its relationship with the recovery of oxygenation, successful liberation from mechanical ventilation, and 28-day clinical outcomes.

## 2 Materials and methods

### 2.1 Study population

This was a prospective, single-center observational cohort study. The study protocol was reviewed and approved by the Institutional Review Board of the participating institution (approval no.YY-LL-2022-041) and conducted in accordance with the principles of the Declaration of Helsinki. Written informed consent was obtained from all enrolled patients or their legal guardians.

Adult patients admitted to the intensive care unit (ICU) within 24 hours and meeting the Third International Consensus Definitions for Sepsis (Sepsis-3) were consecutively enrolled from January 2024 to June 2025. Inclusion criteria were age ≥ 18 years, ICU admission within 24 hours, and a confirmed clinical diagnosis of sepsis or septic shock. Exclusion criteria included known primary immunodeficiency or ongoing immunosuppressive therapy (including long-term immunosuppression following organ transplantation), hematological malignancy, active viral hepatitis, pregnancy, and refusal to participate (**Supplementary Figure 1**).

### 2.2 Clinical data collection

Upon ICU admission, baseline clinical and laboratory variables were documented within the first 24 hours, encompassing demographic characteristics (age, sex, and body mass index [BMI]), primary infection site, causative microorganisms, two validated severity indices (APACHE II and SOFA), and key inflammatory and hematological markers (PCT, CRP, lactate, white blood cell count, and lymphocyte count).Treatment-related data recorded throughout hospitalization included mechanical ventilation, vasopressor use, antibiotic regimen adjustments, corticosteroid administration, and immunomodulator use. Immunomodulator use was defined as subcutaneous thymosin α1 (10 or 15 mg daily) administered during the ICU stay at the treating physician’s discretion per institutional protocol.

Sepsis type refers to the Sepsis-3 category at ICU admission: sepsis was defined as life-threatening organ dysfunction (SOFA ≥2) from a dysregulated host response to infection without persistent hypotension; septic shock as sepsis requiring vasopressors to maintain MAP ≥65 mmHg plus lactate >2 mmol/L despite adequate fluid resuscitation.

### 2.3 BTLA expression measurement

At three predefined time points (within 24 hours of ICU admission [T0], on day 3 [T3], and on day 7 [T7]), 3 mL of peripheral venous blood was drawn into EDTA-anticoagulated tubes and processed no later than 2 hours after collection. Whole blood (50 μL) was stained with BV570 anti-CD45 (clone HI30), FITC anti-CD3 (HIT3a), APC/Cy7 anti-CD4 (RPA-T4), PE/Cy7 anti-CD8 (SK1), and PE/Dazzle 594 anti-CD272/BTLA (MIH26; all BioLegend, San Diego, CA, USA). Erythrocytes were lysed with BD FACS™ Lysing Solution, and samples were acquired on a BD FACSCanto™ II cytometer (BD Biosciences) with daily CS&T bead verification and CompBead-based compensation at the start of each batch. Analyses were performed in FlowJo v10 using the following sequential gating strategy: lymphocytes (FSC-A/SSC-A) → single cells (FSC-A/FSC-H) → CD3^+^ T cells → CD4^+^ and CD8^+^ subpopulations → BTLA^+^ (**Supplementary Figure 2**). The BTLA-positivity threshold was defined relative to matched isotype controls (PE/Dazzle 594 mouse IgG2a, κ) run in parallel for each sample and each batch. BTLA expression was reported as the proportion of BTLA-positive cells within the CD4^+^ and CD8^+^ T cell populations, respectively; MFI/gMFI was not extracted. Fluorescence-minus-one (FMO) controls and viability staining were not performed.

### 2.4 Outcome definitions

The primary outcome was ICU-acquired infection—a new infection arising >48 hours after ICU admission, either at a site distinct from the admitting diagnosis or caused by a pathogen not isolated and not incubating at admission. Suspected events were adjudicated by two board-certified attending physicians in critical care medicine (associate chief physician rank or above), both blinded to BTLA results, using clinical, imaging, and microbiological data. Consensus was required, with disagreements resolved by a third arbiter of equivalent seniority. Infections judged to reflect progression of the index infection were excluded. Infection types were classified according to current diagnostic criteria, including ventilator-associated pneumonia (VAP) (Kalil et al., 2016), catheter-related bloodstream infection (CRBSI) (Mermel et al., 2009), catheter-associated urinary tract infection (CAUTI) (Hooton et al., 2010), and intra-abdominal infection (IAI) (Solomkin et al., 2010). Secondary outcomes included pulmonary recovery indicators (serial PaO_2_/FiO_2_ ratio, time to ventilator weaning, and 28-day ventilator-free days [VFD]), ICU length of stay, and 28-day all-cause mortality. Successful weaning was defined per the WIND framework (Béduneau et al., 2016) as ≥48 consecutive hours off invasive ventilation without reintubation or death. Patients planned for post-extubation non-invasive ventilation were included if reintubation-free for ≥48 h. Reintubation within 48 h counted as failure, with success reassigned to a later successful extubation if one occurred. Tracheostomy for prolonged ventilatory support was treated as failure to wean from invasive ventilation and handled as a competing event: censored at the date of tracheostomy in the cause-specific Cox model, and modeled explicitly as a competing event in the Fine–Gray model. Death before successful weaning and withdrawal of life-sustaining treatment were likewise treated as competing events in both models.

### 2.5 Statistical analysis

Using the median CD4^+^ T cell BTLA expression level within 24 hours of ICU admission as a threshold, patients were categorized into high- and low-expression groups. Because no established clinical cut-point exists for T cell BTLA expression, the median was used to define groups for descriptive comparison and survival visualization only. In all regression analyses BTLA expression was modeled as a continuous variable to avoid loss of information. CD4^+^ T cell BTLA was selected as the primary analytical marker on the basis of its greater between-group separation and comparable discriminative capacity for ICU-acquired infection relative to CD8^+^ T cell BTLA. This focus also limited the number of simultaneous statistical comparisons relative to the available sample size. Continuous variables were summarized as mean ± standard deviation or median (interquartile range) based on normality testing, and compared using the independent samples t-test or Mann–Whitney U test. Categorical variables were presented as frequencies and percentages and compared using the chi-squared test or Fisher’s exact test. Multivariable binary logistic regression was applied to examine the independent association between BTLA expression and ICU-acquired infection risk, with results reported as odds ratios (OR) with 95% confidence intervals (CI). To respect the limited number of events per variable in this cohort, a parsimonious, prespecified covariate set was used, with APACHE II retained as the single composite severity index given its collinearity with SOFA. The infection model was adjusted for APACHE II score and mechanical ventilation. Time to successful ventilator weaning was analyzed within a competing-risks framework in which death before weaning was treated as a competing event rather than as censoring. Cumulative incidence functions were compared between groups using Gray’s test, and the association with admission BTLA expression was estimated using both a cause-specific Cox proportional hazards model (with death censored) and a Fine–Gray subdistribution hazard model, each adjusted for APACHE II score, baseline PaO_2_/FiO_2_ ratio, and sepsis type. As a complementary respiratory outcome that incorporates mortality by assigning zero days to non-survivors, 28-day ventilator-free days (Brower et al., 2000) were compared using the Mann–Whitney U test. Longitudinal changes in BTLA expression were analyzed using a linear mixed-effects model with patient-level random intercepts to evaluate group-by-time interaction effects. ROC curves and the corresponding AUC were constructed to evaluate the discriminative capacity of BTLA for infection outcomes, with the optimal threshold identified via the Youden index.

Missing longitudinal measurements at day 7 (**Supplementary Table 1**) were handled using the maximum-likelihood estimation of the linear mixed-effects model, which provides unbiased estimates under a missing-at-random (MAR) assumption.

Given the limited number of deaths, the analysis of 28-day mortality was considered exploratory and adjusted for APACHE II score only. All analyses were performed using R software (version 4.5.2), with a two-sided P < 0.05 considered statistically significant.

## 3 Results

### 3.1 Baseline characteristics

In total, 143 sepsis patients were classified into a high-expression group (n = 72) and a low-expression group (n = 71) based on median CD4^+^ T cell BTLA expression at admission (50.24 ± 4.63% vs. 36.45 ± 4.10%; P < 0.001). BTLA expression on CD8^+^ T cells was similarly elevated in the high-expression group (41.12 ± 6.78% vs. 34.37 ± 6.75%; P < 0.001). Baseline demographics, including age, sex, BMI, and primary infection site distribution, were comparable between groups (P > 0.05). The high-expression group demonstrated substantially greater illness severity, with higher APACHE II scores (22.92 ± 4.88 vs. 13.17 ± 4.60; P < 0.001), SOFA scores (7.42 ± 2.48 vs. 5.15 ± 2.64; P < 0.001), and a higher proportion of septic shock (94.4% vs. 38.0%; P < 0.001). Laboratory parameters were also significantly worse, including PCT (20.57 ± 5.57 ng/mL vs. 9.26 ± 5.83 ng/mL; P < 0.001), CRP (174.06 ± 50.20 mg/L vs. 116.17 ± 44.50 mg/L; P < 0.001), lactate (5.96 ± 2.10 mmol/L vs. 3.15 ± 1.48 mmol/L; P < 0.001), and PaO_2_/FiO_2_ ratio (150.99 ± 55.69 mmHg vs. 271.13 ± 104.35 mmHg; P < 0.001). White blood cell and lymphocyte counts did not differ significantly between groups (P > 0.05). Rates of mechanical ventilation (94.4% vs. 56.3%; P < 0.001) and vasopressor use (93.1% vs. 49.3%; P < 0.001) were higher in the high-expression group, whereas antibiotic adjustment, corticosteroid use, immunomodulator use, and CRRT did not differ significantly (all P > 0.05). Clinical outcomes were significantly worse in the high-expression group, with a longer ICU stay (16.65 ± 4.99 days vs. 10.21 ± 4.03 days; P < 0.001), higher 28-day mortality (33.3% vs. 9.9%; P = 0.001), and a higher ICU-acquired infection rate (38.9% vs. 21.1%; P = 0.033). Complete baseline characteristics are presented in **Table 1**.

**Table 1:** Baseline characteristics of sepsis patients stratified by BTLA expression on CD4^+^ T cells.

Variable	Low BTLA-CD4^+^ (n = 71)	High BTLA-CD4^+^ (n = 72)	P-value
Demographics			
Age, years (mean ± SD)	72.46 (11.02)	71.94 (10.13)	0.769
Sex, male (%)	35 (49.3)	39 (54.2)	0.617
Body mass index, kg/m^2^ (mean ± SD)	23.24 ± 3.50	23.95 ± 3.47	0.222
Primary infection site, n (%)			
* Abdomen*	25 (35.2)	12 (16.7)	0.129
* Bloodstream*	12 (16.9)	14 (19.4)	
* Lung*	23 (32.4)	30 (41.7)	
* Skin*	2 (2.8)	5 (6.9)	
* Urinary tract*	9 (12.7)	11 (15.3)	
Sepsis type, n (%)			
* Sepsis*	44 (62.0)	4 (5.6)	<0.001
* Septic shock*	27 (38.0)	68 (94.4)	
Disease severity			
APACHE II score (mean ± SD)	13.17 ± 4.60	22.92 ± 4.88	<0.001
SOFA score (mean ± SD)	5.15 ± 2.64	7.42 ± 2.48	<0.001
Laboratory parameters			
Procalcitonin, ng/mL (mean ± SD)	9.26 ± 5.83	20.57 ± 5.57	<0.001
C-reactive protein, mg/L (mean ± SD)	116.17 ± 44.50	174.06 ± 50.20	<0.001
Lactate, mmol/L (mean ± SD)	3.15 ± 1.48	5.96 ± 2.10	<0.001
White blood cell count, ×10^9^/L (mean ± SD)	12.23 ± 5.05	13.00 ± 4.85	0.357
Lymphocyte count, ×10^9^/L (mean ± SD)	0.72 ± 0.28	0.75 ± 0.30	0.576
BTLA expression			
BTLA on CD4^+^ T cells, % (mean ± SD)	36.45 ± 4.10	50.24 ± 4.63	<0.001
BTLA on CD8^+^ T cells, % (mean ± SD)	34.37 ± 6.75	41.12 ± 6.78	<0.001
PaO_2_/FiO_2_ ratio, mmHg (mean ± SD)	271.13 ± 104.35	150.99 ± 55.69	<0.001
Clinical interventions, n (%)			
Mechanical ventilation	40 (56.3)	68 (94.4)	<0.001
Vasopressor use	35 (49.3)	67 (93.1)	<0.001
Antibiotic adjustment	41 (57.7)	45 (62.5)	0.682
Corticosteroid use	39 (54.9)	32 (44.4)	0.277
Immunomodulator use	58 (81.7)	53 (73.6)	0.338
Continuous renal replacement therapy	14 (19.7)	20 (27.8)	0.327
Clinical outcomes			
ICU length of stay, days (mean ± SD)	10.21 ± 4.03	16.65 ± 4.99	<0.001
28-day mortality, n (%)	7 (9.9)	24 (33.3)	0.001
ICU-acquired infection, n (%)	15 (21.1)	28 (38.9)	0.033

Data are presented as mean ± standard deviation (SD) or number (percentage).Abbreviations: BTLA, B and T lymphocyte attenuator; APACHE II, Acute Physiology and Chronic Health Evaluation II; SOFA, Sequential Organ Failure Assessment; PCT, procalcitonin; CRP, C-reactive protein; WBC, white blood cell; PaO_2_/FiO_2_, arterial oxygen partial pressure to fractional inspired oxygen; ICU, intensive care unit.

### 3.2 Association Between BTLA Expression and ICU-Acquired Infection

**Figure 1:**
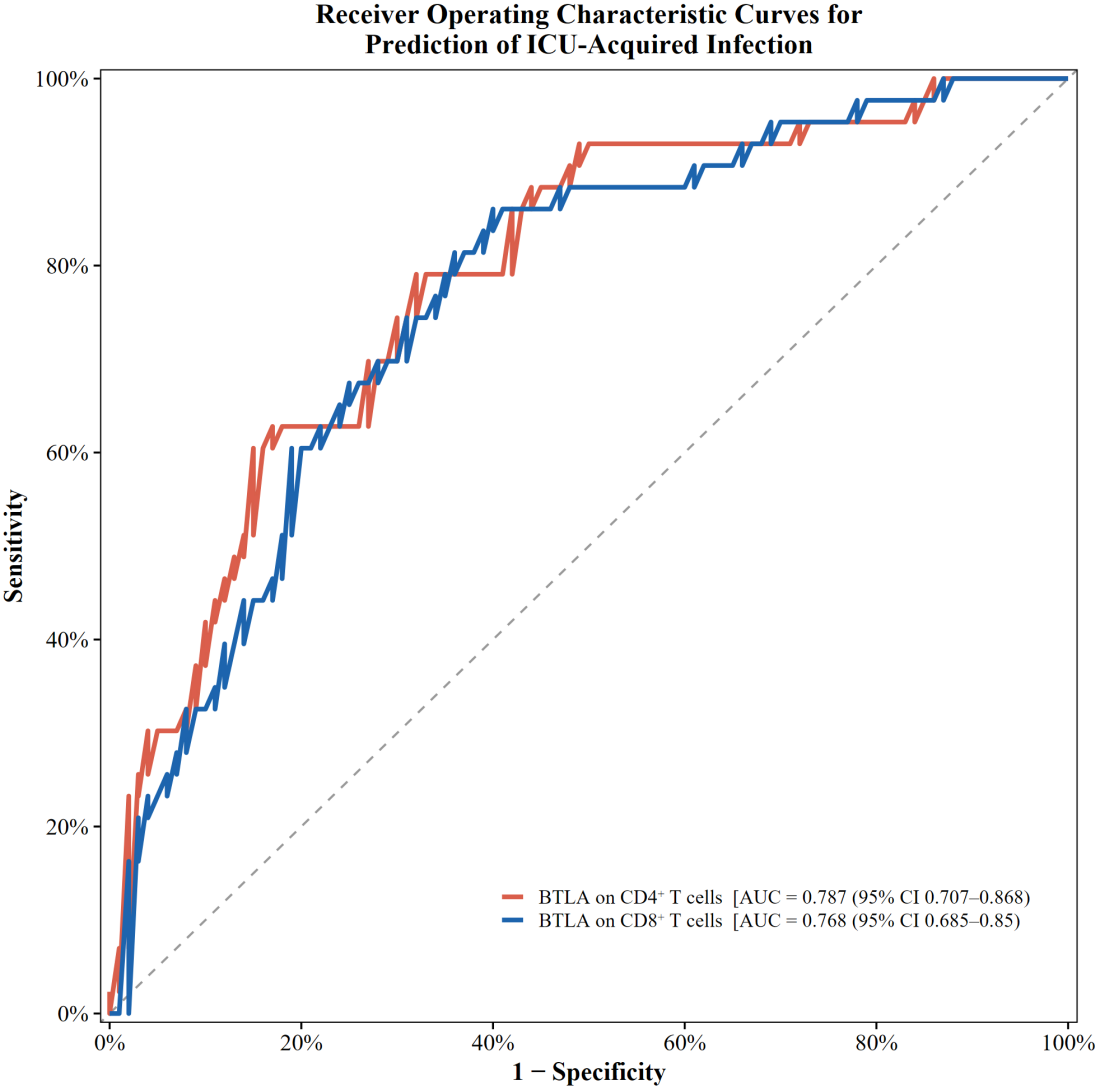
Receiver operating characteristic curves for prediction of ICU-acquired infection.

Upon blinded adjudication, 43 patients (30.1%) were confirmed to have developed ICU-acquired infection, with the high-expression group exhibiting a substantially greater incidence than the low-expression group (38.9% vs. 21.1%; P = 0.033). Detailed characteristics of the 43 events (type, timing, microbiological confirmation, and new-site status) are provided in **Supplementary Table 2**. VAP and CRBSI constituted the most frequently encountered infection types. As illustrated in **Figure 1**, ROC curve analysis revealed moderate discriminative capacity of BTLA-CD4 for identifying ICU-acquired infection (AUC = 0.787, 95% CI: 0.707–0.868), while BTLA-CD8 yielded a comparable AUC of 0.768 (95% CI: 0.685–0.850).

Following multivariable adjustment for APACHE II score and mechanical ventilation, BTLA-CD4 expression was not independently associated with ICU-acquired infection (adjusted OR = 0.937, 95% CI: 0.859–1.021, P = 0.139). In contrast, APACHE II score remained an independent predictor of infection (OR = 1.199, 95% CI: 1.068–1.346, P = 0.002) (**Table 2**, Model 1).

**Table 2:** Multivariable regression analyses of the independent associations between BTLA expression on CD4^+^ T cells and ICU-acquired infection risk, ventilator weaning, and 28-day mortality.

Variable	OR, HR, or sHR (95% CI)	P-value
Model 1: ICU-acquired infection (multivariable logistic regression, n = 143)		
**BTLA expression on CD4^+^ T cells (per 1%)**	0.937 (0.859–1.021)	0.139
APACHE II score	1.199 (1.068–1.346)	0.002
Mechanical ventilation	2.353 (0.557–9.939)	0.244
Model 2a: Time to ventilator weaning, cause-specific Cox model *(death censored; mechanically ventilated patients, n = 108)*		
**BTLA expression on CD4^+^ T cells (per 1%)**	0.882 (0.832–0.936)	<0.001
APACHE II score	0.874 (0.788–0.971)	0.012
Baseline PaO_2_/FiO_2_ ratio	0.999 (0.994–1.004)	0.607
Septic shock (ref: sepsis)	1.129 (0.480–2.652)	0.781
Model 2b: Time to ventilator weaning, Fine–Gray subdistribution hazard model *(death as competing event; n = 108)*		
**BTLA expression on CD4^+^ T cells (per 1%)**	0.940 (0.902–0.981)	0.004
APACHE II score	0.959 (0.877–1.048)	0.353
Baseline PaO_2_/FiO_2_ ratio	1.006 (1.001–1.010)	0.016
Septic shock (ref: sepsis)	2.797 (1.245–6.285)	0.013
Model 3 (exploratory): 28-day all-cause mortality *(multivariable Cox regression, n = 143)*		
**BTLA expression on CD4^+^ T cells (per 1%)**	1.034 (0.965–1.108)	0.342
APACHE II score	1.086 (0.996–1.185)	0.063

OR, odds ratio; HR, hazard ratio; sHR, subdistribution hazard ratio; CI, confidence interval; BTLA, B and T lymphocyte attenuator; APACHE II, Acute Physiology and Chronic Health Evaluation II; PaO_2_/FiO_2_, arterial oxygen partial pressure to fractional inspired oxygen. All models used BTLA on CD4^+^ T cells as a continuous variable (per 1% increase) and, to respect the number of events per variable, a parsimonious covariate set with APACHE II as the single severity index. Model 1, multivariable logistic regression for ICU-acquired infection (n = 143). Models 2a and 2b, competing-risks analyses of time to successful ventilator weaning in mechanically ventilated patients (n = 108), with death before weaning treated as a competing event: Model 2a, cause-specific Cox model (death censored); Model 2b, Fine–Gray subdistribution hazard model. Model 3 (exploratory), Cox model for 28-day all-cause mortality (n = 143). Bold rows indicate the primary exposure variable (BTLA expression on CD4^+^ T cells).

### 3.3 Longitudinal dynamics of BTLA expression

**Figure 2:**
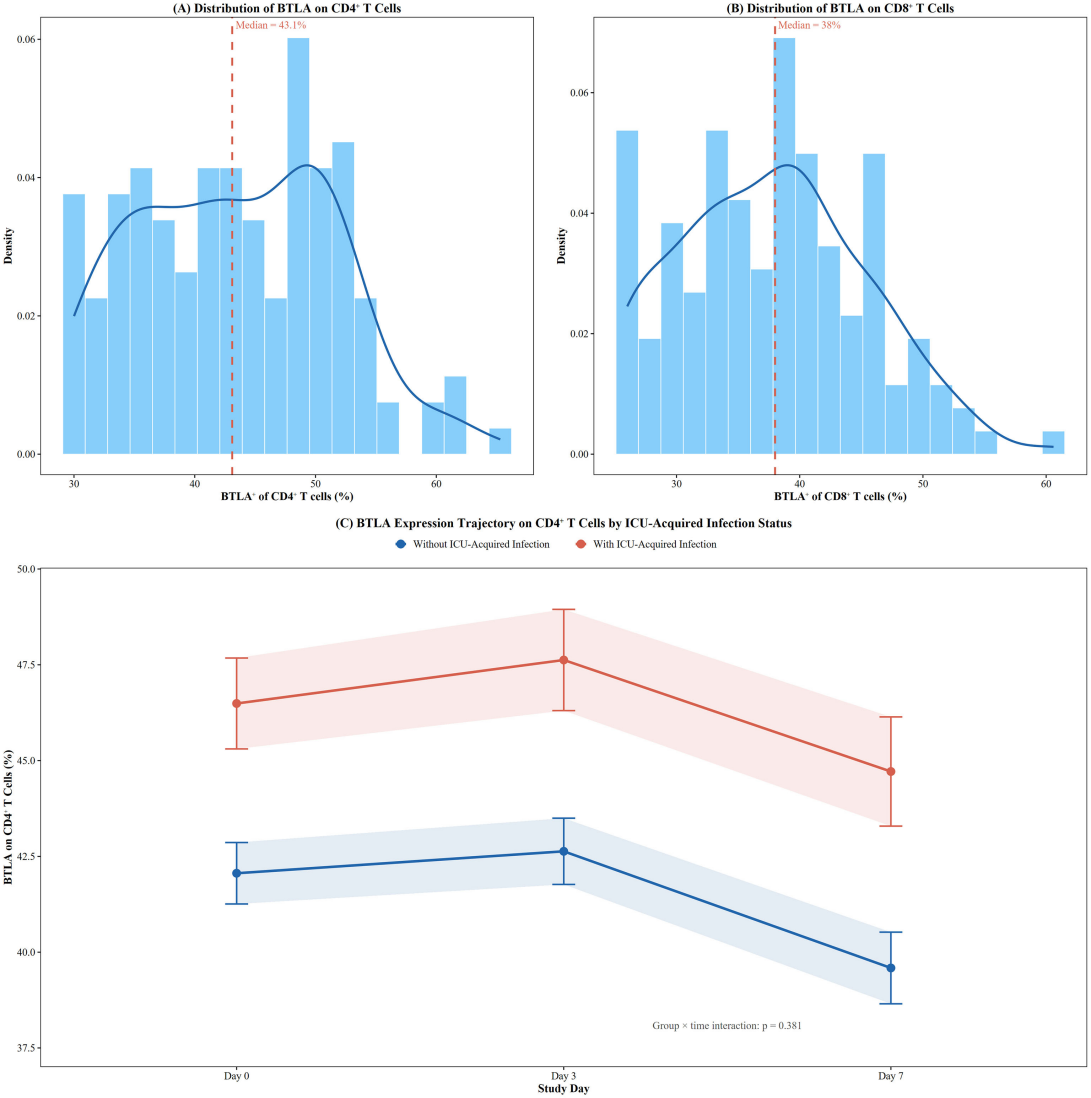
Distribution of BTLA expression on T cells and its longitudinal trajectory. **(A, B)** Distribution of the percentage of BTLA-positive cells among CD4^+^ **(A)** and CD8^+^ **(B)** T cells at ICU admission across the cohort. **(C)** Longitudinal trajectory of BTLA expression on CD4^+^ T cells stratified by ICU-acquired infection status (mean ± standard error).

BTLA expression on CD4^+^ T cells was consistently higher in patients with ICU-acquired infection than in those without across all three time points (Day 0: 46.7 ± 1.1% vs. 41.9 ± 0.8%; Day 3: 47.8 ± 1.3% vs. 42.5 ± 0.9%; Day 7: 45.4 ± 1.3% vs. 39.7 ± 1.0%). Regarding longitudinal trends, BTLA expression declined from Day 3 to Day 7 in patients without ICU-acquired infection, whereas it remained persistently elevated in patients with ICU-acquired infection. The group-by-time interaction was not statistically significant in the linear mixed-effects model (P = 0.381), indicating that the between-group difference reflected a persistently higher level of expression rather than a divergent temporal trajectory. These findings are illustrated in **Figure 2**. The number of participants contributing BTLA and PaO2/FiO2 measurements at each time point, stratified by BTLA group and ICU-acquired infection status, is summarized in **Supplementary Table 1**.

### 3.4 Association between BTLA expression and time to ventilator weaning

**Figure 3:**
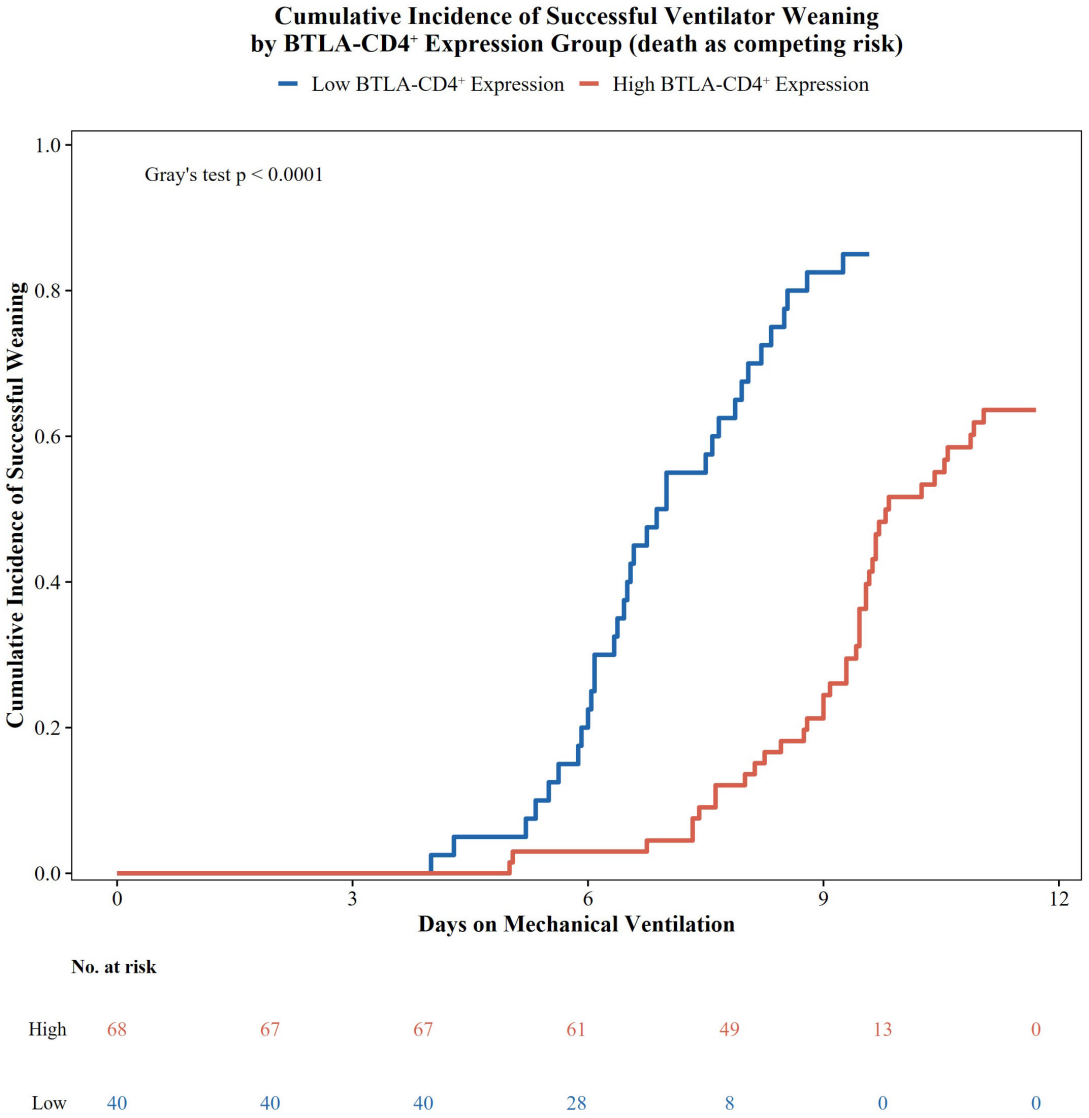
Cumulative incidence of successful ventilator weaning stratified by admission BTLA expression on CD4^+^ T cells.

Among the 108 mechanically ventilated patients, the cumulative incidence of successful weaning, with death before weaning treated as a competing event, was significantly higher in the low-expression group (Gray’s test, P < 0.001). By day 7, the cumulative incidence of successful weaning was 55.0% in the low-expression group versus 4.5% in the high-expression group. By day 14 it was 85.0% versus 63.6%, respectively (**Figure 3**). In a cause-specific Cox model adjusted for APACHE II score, baseline PaO_2_/FiO_2_ ratio, and sepsis type, higher admission BTLA-CD4 expression was independently associated with a lower rate of successful weaning (HR = 0.882 per 1% increment, 95% CI: 0.832–0.936, P < 0.001; equivalent to HR = 0.286 per 10% increment, 95% CI: 0.158–0.515, P < 0.001) (**Table 2**, Model 2a). APACHE II score was also independently associated with weaning rate in this model (HR = 0.874, 95% CI: 0.788–0.971, P = 0.012). The association between BTLA-CD4 expression and weaning rate persisted after accounting for the competing risk of death in a Fine–Gray subdistribution hazard model (sHR = 0.940, 95% CI: 0.902–0.981, P = 0.004) (**Table 2**, Model 2b). In the Fine–Gray model, baseline PaO_2_/FiO_2_ ratio (sHR = 1.006, 95% CI: 1.001–1.010, P = 0.016) and septic shock (sHR = 2.797, 95% CI: 1.245–6.285, P = 0.013) were also independently associated with weaning (**Table 2**, Model 2b).

### 3.5 Association between BTLA expression and respiratory function recovery

**Figure 4:**
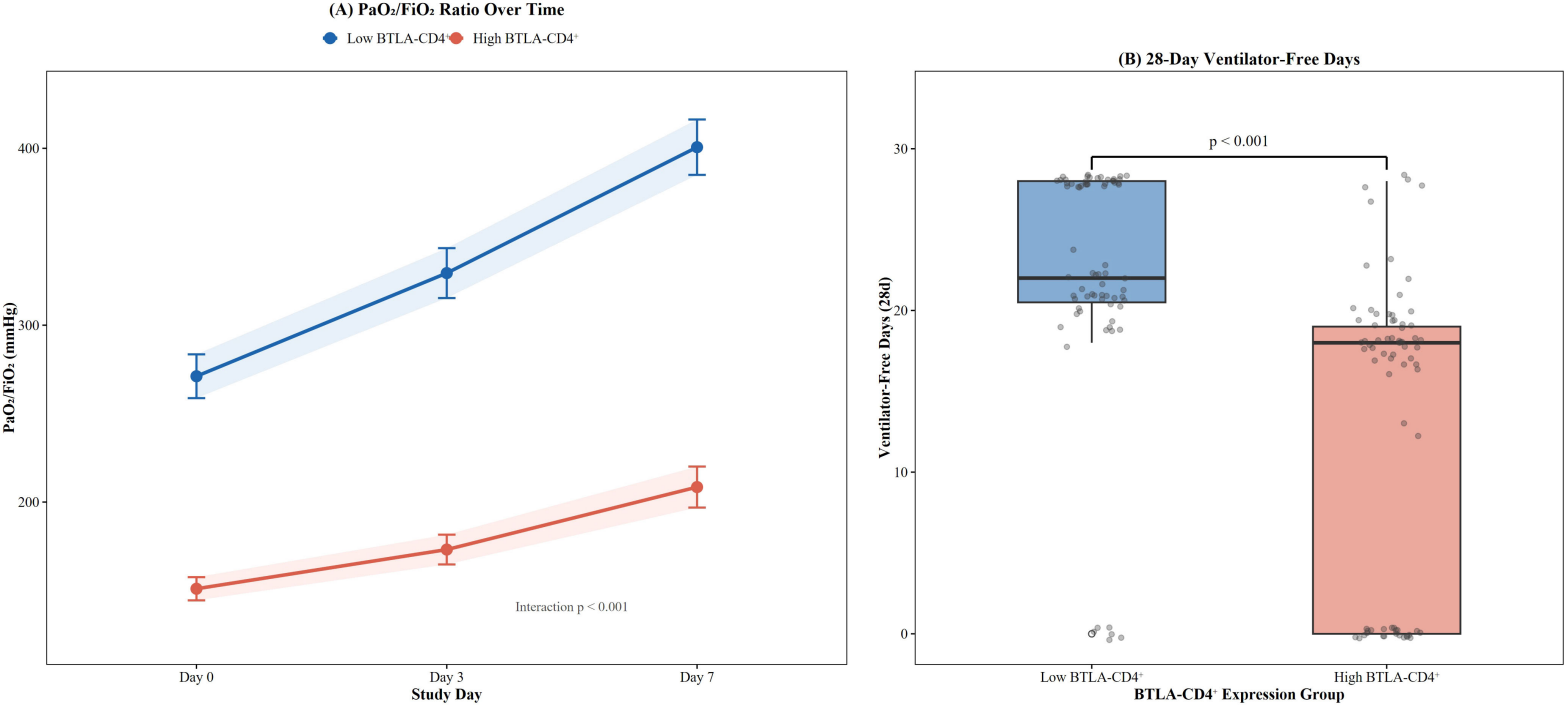
Comparison of pulmonary recovery indicators between groups stratified by BTLA expression level on CD4^+^ T cells. **(A)** Dynamic changes in PaO_2_/FiO_2_ ratio at T0, T3, and T7 (mean ± standard error). **(B)** 28-day ventilator-free days between groups.

Both groups exhibited progressive improvement in PaO_2_/FiO_2_ ratios over the observation period. However, oxygenation levels remained consistently superior in the low-expression group across all time points (P < 0.001). A statistically significant group-by-time interaction was detected by the linear mixed-effects model (P < 0.001), suggesting that oxygenation recovery proceeded at a faster pace in the low-expression group, with the between-group disparity progressively widening over time (**Figure 4A**).

### 3.6 28-day ventilator-free days

Patients in the low-expression group accumulated significantly more 28-day ventilator-free days relative to those in the high-expression group (P < 0.001). A notable subset of high-expression patients recorded zero ventilator-free days, largely attributable to in-hospital death during mechanical ventilation or inability to achieve successful weaning within the 28-day window, collectively indicating a poorer respiratory outcome in this subgroup (**Figure 4B**).

### 3.7 ICU length of stay and 28-day survival

ICU length of stay was longer in the high-expression group than in the low-expression group (16.65 ± 4.99 days vs. 10.21 ± 4.03 days; P < 0.001). Among the two subgroups, 28-day mortality was higher in the high-expression group than in the low-expression group (33.3% vs. 9.9%; P = 0.001) (**Table 1**). However, in an exploratory Cox model adjusted for APACHE II score–limited by the small number of deaths–BTLA-CD4 expression was not independently associated with 28-day mortality (HR = 1.034, 95% CI: 0.965–1.108, P = 0.342), and APACHE II score did not reach statistical significance in this model (HR = 1.086, 95% CI: 0.996–1.185, P = 0.063). Taken together, these findings indicate that the excess mortality noted in the high-expression group is more likely a reflection of greater underlying disease severity than a direct consequence of BTLA expression on mortality outcomes (**Table 2**, Model 3).

## 4 Discussion

Through longitudinal profiling of BTLA expression on CD4^+^ T cells in critically ill septic patients, this prospective study comprehensively examined its relationships with ICU-acquired infection, ventilator liberation, and 28-day clinical outcomes. Elevated BTLA expression was linked to a greater incidence of ICU-acquired infection, and CD4^+^ T cell BTLA expression was independently associated with prolonged weaning from mechanical ventilation, even after accounting for the competing risk of death. Collectively, these observations imply that the clinical significance of BTLA in sepsis reaches beyond its utility as an infection risk marker and is associated with impaired respiratory recovery and delayed ventilator liberation.

The relationship identified between elevated CD4^+^ T cell BTLA expression and higher ICU-acquired infection rates aligns with prior evidence in the field. An earlier prospective investigation documented a positive correlation between upregulated BTLA expression on CD4^+^ T cells and the subsequent emergence of secondary nosocomial infections among septic patients (Shubin et al., 2013). Comparable observations have similarly been reported in the context of *Candida* sepsis (Spec et al., 2016). However, this association did not remain significant after multivariable adjustment in the present study, suggesting that the incremental predictive contribution of elevated BTLA expression to infection risk may not be independent of the background of disease severity. Notably, patients in the high-expression group exhibited higher baseline APACHE II scores, SOFA scores, and levels of PCT, CRP, and serum lactate, indicating that elevated BTLA co-exists with more severe systemic inflammatory responses and organ dysfunction. Accordingly, the clinical utility of BTLA as a predictor of infection may be best realized through its integration with complementary immunological biomarkers (Venet and Monneret, 2018).

Serial assessment of BTLA expression across multiple time points revealed that BTLA levels on CD4^+^ T cells remained persistently elevated throughout the observation period in patients who developed ICU-acquired infection, whereas a declining trend was observed by day 7 in patients without ICU-acquired infection. This longitudinal pattern is consistent with the established dynamics of sepsis-induced immunosuppression: prior longitudinal studies have shown that co-inhibitory molecule expression on T cells declines progressively within the first week in survivors, while remaining persistently elevated in non-survivors or patients with protracted illness (Boomer et al., 2012). Given that sustained high expression of PD-1 on T cells has been independently associated with increased 28-day mortality in sepsis (Guignant et al., 2011), the similar longitudinal trajectory of BTLA observed here suggests that BTLA may likewise contribute to the perpetuation of T cell exhaustion in this setting.

Of particular note among the present study’s findings is the independent association between CD4^+^ T cell BTLA expression and time to ventilator liberation. After accounting for the competing risk of death and adjusting for disease severity, baseline oxygenation, and sepsis type, both cause-specific and Fine–Gray subdistribution hazard models confirmed BTLA-CD4 as an independent determinant of delayed weaning. To the best of our knowledge, no previous investigation has directly addressed the link between T cell co-inhibitory molecule expression and weaning outcomes, rendering the current study the first to offer clinical evidence in this context. Consistent with this finding, the low-expression group exhibited markedly more rapid improvement in PaO_2_/FiO_2_ ratios over time relative to high-expression counterparts, with the between-group disparity progressively widening throughout the observation period. Whether elevated CD4^+^ T cell BTLA expression contributes causally to delayed pulmonary recovery, or simply marks a broader immune-suppressive phenotype associated with impaired recovery, cannot be determined from the present data. We did not directly measure T cell function, BTLA/HVEM signaling, alveolar injury, cytokine production, or the response to BTLA pathway modulation. Prior work has shown that adaptive immune responses regulate the resolution of sepsis-associated ARDS (Matthay et al., 2019) and that CD4^+^CD25^+^ regulatory T cells are required for the resolution phase of experimental acute lung injury (D'Alessio et al., 2009), but these mechanistic links cannot be extrapolated to establish a causal role for BTLA in the present cohort. Our findings should therefore be interpreted as showing that elevated CD4^+^ T cell BTLA expression at admission identifies a sepsis immune phenotype associated with impaired respiratory recovery, rather than as evidence that BTLA regulates lung repair.

Regarding survival outcomes, despite a significantly greater 28-day mortality rate in the high-expression group on univariable analysis, BTLA-CD4 expression did not independently predict 28-day mortality after adjustment for illness severity. This mortality analysis was exploratory given the limited number of deaths. This pattern mirrors the findings from the infection analyses and collectively suggests that the univariable association between elevated BTLA expression and adverse outcomes is largely attributable to the greater underlying disease burden in this group, rather than to an independent effect of BTLA expression per se. This finding differs from reports demonstrating that PD-1 independently predicts sepsis mortality (Guignant et al., 2011), a discrepancy that may reflect the more complex bidirectional signaling mediated by the BTLA/HVEM axis, whose inhibitory effects on T cell function may be less direct than those of the PD-L1 pathway (Murphy and Murphy, 2010).

Several limitations of this study warrant acknowledgment. The single-center design constrain the generalizability of the findings, which require validation in multicenter cohorts. Second, the BTLA threshold was defined by the median value of the study cohort and lacks external validation, limiting its direct clinical applicability. However, because all regression analyses modeled BTLA as a continuous variable, the principal findings do not depend on this threshold. Third, only surface BTLA expression on T cells was assessed, without concurrent measurement of HLA-DR expression, PD-1 co-expression, or cytokine profiles, precluding a comprehensive characterization of the immune landscape. Fourth, although the >48-hour definition and independent blinded adjudication requiring a distinct anatomical site or a new causative pathogen were used to distinguish ICU-acquired infection from progression of the index infection, residual misclassification cannot be entirely excluded. Fifth, the observational design cannot fully exclude residual confounding, and causal inference regarding the association between BTLA expression and weaning outcomes awaits confirmation from interventional studies. Sixth, only CD4^+^ T cell BTLA was carried forward into the principal multivariable analyses. Although BTLA on CD8^+^ T cells yielded a comparable AUC for ICU-acquired infection, its independent contribution to the outcomes examined here remains to be characterized in future studies with larger sample sizes. Seventh, BTLA-positivity thresholds were defined using isotype rather than fluorescence-minus-one (FMO) controls, viability staining was not performed, and BTLA was analyzed only as percent-positive cells without MFI/gMFI extraction. Because the same thresholding approach was applied uniformly across all samples, time points, and groups, differential misclassification between groups is unlikely to affect the direction of the reported associations. Finally, missingness at day 7 resulted entirely from death before that time point, and such missingness is unlikely to be completely at random. While the linear mixed-effects model assumes missingness at random, missing-not-at-random cannot be excluded, and the observed trajectories may therefore underestimate the burden in the sickest patients.

## 5 Conclusion

The findings of this study suggest that elevated BTLA expression on CD4^+^ T cells in sepsis predominantly reflects a composite marker of immune suppression and disease severity, as its predictive value for ICU-acquired infection and 28-day mortality was not maintained after adjustment for illness acuity. In contrast, elevated CD4^+^ T cell BTLA expression at admission was independently associated with delayed liberation from mechanical ventilation, identifying a sepsis immune phenotype associated with impaired respiratory recovery. Future investigations should focus on validating these findings in large multicenter cohorts, exploring integrated immunological assessment models combining BTLA with HLA-DR, PD-1, and other immune biomarkers.

## Data Availability

The original contributions presented in the study are included in the article/**Supplementary Material**. Further inquiries can be directed to the corresponding author.
