# Supplementary material for: Admission CD4+ T cell BTLA expression and its association with infection risk and respiratory outcomes in sepsis
Source: Front Cell Infect Microbiol. 2026 Aug 3;16:1852916. doi: 10.3389/fcimb.2026.1852916 (PMC13477970; doi:10.3389/fcimb.2026.1852916)
Supplement: Supplementary file 1 [file SupplementaryFile1.docx]

**Supplementary Materials**

**1. Supplementary Tables**

**Supplementary Table S1.** Available BTLA and PaO₂/FiO₂ measurements at each time point.

| Time point / Variable | Low BTLA-CD4 | High BTLA-CD4 | With ICU-acquired infection (n = 43) | Without ICU-acquired infection (n = 100) | Total (n = 143) |
| --- | --- | --- | --- | --- | --- |
| **T0 (admission, within 24 h)** | |  |  |  |  |
| BTLA-CD4 available | 72 | 71 | 43 | 100 | 143 |
| PaO₂/FiO₂ available | 72 | 71 | 43 | 100 | 143 |
| **T3 (day 3)** | | |  |  |  |
| BTLA-CD4 available | 72 | 71 | 43 | 100 | 143 |
| PaO₂/FiO₂ available | 72 | 71 | 43 | 100 | 143 |
| **T7 (day 7)** | |  |  |  |  |
| BTLA-CD4 available | 71 | 67 | 40 | 98 | 138 |
| PaO₂/FiO₂ available | 71 | 67 | 40 | 98 | 138 |
| Missing – death before day 7 | 1 | 4 | 3 | 2 | 5 |
| Missing – ICU discharge before day 7 | 0 | 0 | 0 | 0 | 0 |
| Missing – failed sampling / other | 0 | 0 | 0 | 0 | 0 |

**Notes:** BTLA-CD4, B and T lymphocyte attenuator expression on CD4⁺ T cells; PaO₂/FiO₂, arterial oxygen partial pressure to fractional inspired oxygen ratio. Low- and High-BTLA groups were defined by the median admission BTLA-CD4 expression (43.1%). Patients with vs. without ICU-acquired infection are shown as separate stratifications of the same 143-patient cohort. All 5 patients with missing T7 measurements died before day 7; death dates ranged from day 4 to day 6 relative to ICU admission. Missing longitudinal measurements were handled under the maximum-likelihood framework of the linear mixed-effects model.

**Supplementary Table S2.** Details of ICU-acquired infection events (n = 43).

| Infection type | n | Days from ICU admission, median (IQR) | Microbiological confirmation | New site relative to primary infection |
| --- | --- | --- | --- | --- |
| VAP | 21 | 8 (5–11) | Culture-confirmed: 18/21 (86%); clinical + radiographic only: 3/21 (14%) | New site: 14/21 (67%); same site with new pathogen: 7/21 (33%) |
| CRBSI | 12 | 12 (9–12) | Blood culture-positive: 12/12 (100%) | New site: 9/12 (75%); same site with new pathogen: 3/12 (25%) |
| CAUTI | 5 | 11 (9–13) | Urine culture-positive: 5/5 (100%) | New site: 5/5 (100%) |
| IAI | 5 | 6 (5–7) | Culture-confirmed: 4/5 (80%); imaging + clinical only: 1/5 (20%) | New site: 4/5 (80%); same site with new pathogen: 1/5 (20%) |
| All types | 43 | 9 (6–12) | Culture-confirmed: 39/43 (91%) | New site: 32/43 (74%); same site with new pathogen: 11/43 (26%) |

**Notes:** VAP, ventilator-associated pneumonia; CRBSI, catheter-related bloodstream infection; CAUTI, catheter-associated urinary tract infection; IAI, intra-abdominal infection; IQR, interquartile range. Days from ICU admission are calculated from the date of the T0 sample to the date of infection onset. Culture confirmation refers to a positive culture from a clinically relevant specimen (respiratory secretions, blood, urine, or peritoneal fluid/drainage). Cases classified as "clinical + radiographic only" or "imaging + clinical only" were adjudicated on the basis of clinical presentation and imaging findings in the absence of culture confirmation.

**2. Supplementary Figures**

**Supplementary Figure S1.** Study flow diagram.

**Supplementary Figure S2.** Representative gating strategy for BTLA⁺ CD4⁺ and CD8⁺ T cells.
